# Association between fluid balance trajectories and prognosis in older patients with malnutrition: a group-based trajectory model analysis

**DOI:** 10.3389/fnut.2026.1796378

**Published:** 2026-04-15

**Authors:** Chunmei Zhang, Zhiyi Xie, Qitian Zhang, Xiaohong Huang

**Affiliations:** Department of Cardiology, Zhangzhou Affiliated Hospital of Fujian Medical University, Zhangzhou, Fujian, China

**Keywords:** fluid balance, group-based trajectory model, MIMIC database, mortality risk, older malnutrition, prognosis

## Abstract

**Background:**

Fluid balance plays a crucial role in the management of older patients with malnutrition; however, the impact of different fluid balance trajectories on prognosis has not been fully elucidated. This study aimed to evaluate the association between fluid balance trajectories and the risk of 30-day mortality in older patients with malnutrition.

**Methods:**

This study was conducted using data from the Medical Information Mart for Intensive Care IV (MIMIC-IV) database. Patients diagnosed with malnutrition among the older population were included. A group-based trajectory model (GBTM) was applied to identify subgroups of patients with similar trends in fluid balance (FB). Kaplan–Meier survival analysis and Cox proportional hazards regression models were used to assess the associations between different fluid balance trajectories and patient survival. In addition, subgroup analyses and sensitivity analyses were performed to evaluate the robustness of the findings.

**Results:**

A total of 1,778 older patients with malnutrition were included. Four distinct fluid balance trajectory patterns were identified: trajectory 1 (T1, persistent positive balance), trajectory 2 (T2, mild negative balance), trajectory 3 (T3, high-level rapid decline), and trajectory 4 (T4, moderate-level rapid decline). Kaplan–Meier survival analysis showed that patients in trajectory 1 (persistent positive balance) had the highest 30-day mortality rate (46.2%), whereas those in trajectory 4 (moderate-level rapid decline) had the lowest mortality risk (26.6%). After adjustment for potential confounders, Cox regression analysis further demonstrated that, compared with trajectory 1 as the reference group, trajectory 4 was significantly associated with a lower risk of mortality [hazard ratio (HR) = 0.59, 95% confidence interval (CI): 0.47–0.73].

**Conclusion:**

Fluid balance trajectories were significantly associated with prognosis in older patients with malnutrition, and dynamic patterns of fluid balance may aid in stratifying mortality risk. The GBTM approach effectively identified patient subgroups with different risk profiles, providing valuable insights for clinical fluid management.

## 1 Introduction

Malnutrition is a condition characterized by reduced body composition and impaired physiological function resulting from inadequate nutrient intake or utilization, typically manifested as weight loss, decreased muscle mass and strength, and impaired immune function (1). Older adults are particularly susceptible to malnutrition due to diminished physiological reserves and multimorbidity. In the ICU, stress responses, systemic inflammation, and mechanical ventilation further accelerate muscle loss, rendering critically ill older patients a high-risk population (2). Previous studies have shown that malnutrition is highly prevalent among older ICU patients and is closely associated with increased risks of ICU and in-hospital mortality (3). Malnutrition also increases the risk of infections and complications, prolongs the duration of mechanical ventilation and hospital stay, and adversely affects post-discharge functional recovery and quality of life (4, 5). Moreover, it significantly increases the risk of readmission and healthcare costs, thereby imposing a substantial burden on healthcare systems (6). However, the identification and management of malnutrition in the ICU remain limited by the susceptibility of assessment indicators to inflammation and volume status, as well as insufficient clinical attention (7). Therefore, early identification and standardized management of malnutrition are of great clinical importance.

Fluid balance reflects the relationship between fluid input and output over a given period, with the clinical goal of ensuring adequate organ perfusion while avoiding excessive fluid accumulation (8). In critically ill states, inflammatory responses, increased capillary permeability, and impaired cardiac and renal function promote fluid translocation into the interstitial space, exacerbating pulmonary edema, and multiple organ dysfunction (9). Previous studies have demonstrated that positive or cumulative fluid balance is closely associated with increased disease severity, higher risks of ICU and in-hospital mortality, and prolonged durations of mechanical ventilation and hospitalization (10, 11). However, most existing studies are based on static analyses using single time points or simple cumulative values and primarily focus on the general adult critically ill population. Such approaches fail to capture the dynamic changes in fluid status and overlook the specific characteristics of older and malnourished patients (8, 12). In addition, among older ICU patients, malnutrition, sarcopenia, and reduced physiological reserves may increase susceptibility to fluid overload, yet dynamic investigations in this population remain limited (13). Therefore, more refined, time-oriented studies of fluid balance in critically ill older patients with malnutrition are needed to address existing evidence gaps and optimize individualized management strategies.

The group-based trajectory model (GBTM) is a statistical approach for analyzing longitudinal data that enables population stratification through distinct trajectory patterns and facilitates risk phenotype identification (14). Previous studies have applied GBTM in critical care and clinical research to examine temporal trajectories of various indicators and have demonstrated associations with clinical outcomes (15, 16). However, many existing analyses still rely on static measures derived from single time points or cumulative values, which inadequately reflect true temporal patterns and may underestimate population heterogeneity (17). In older ICU patients with malnutrition, fluid balance exhibits pronounced time dependency and interindividual variability, yet related dynamic studies are scarce. Therefore, this study applied GBTM to identify fluid balance trajectories and evaluate their associations with short-term mortality in this population, providing evidence to support precise risk stratification and early targeted interventions.

## 2 Materials and methods

### 2.1 Data source

This study was conducted using the publicly available critical care database MIMIC-IV(version 3.1). The MIMIC-IV database was jointly developed by the Massachusetts Institute of Technology and Beth Israel Deaconess Medical Center and contains high-quality, de-identified clinical data from more than 65,000 ICU patients (18). The database has received approval from the relevant institutional review boards, with a waiver of informed consent, and all protected health information has been rigorously anonymized. The investigators completed the Collaborative Institutional Training Initiative (CITI) ethics training (Record ID: 73212477) and signed the data use agreement. This study was reported in accordance with the Strengthening the Reporting of Observational Studies in Epidemiology (STROBE) statement (19).

### 2.2 Study population

Older patients with malnutrition admitted to the ICU were included in this study. Malnutrition was defined as a Geriatric Nutritional Risk Index (GNRI) score < 98. The inclusion criteria were as follows: (1) age ≥65 years at ICU admission; (2) availability of GNRI calculation within 24 h after ICU admission with a GNRI < 98; and (3) for patients with multiple ICU admissions, only the first ICU admission during the first hospitalization was included. The exclusion criteria were: (1) ICU length of stay < 3 days; and (2) fewer than three fluid balance measurements within the first 7 days after ICU admission.

GNRI was calculated using the following formula: GNRI = 1.489 × serum albumin (g/L) + 41.7 × (actual body weight / ideal body weight). When the actual body weight exceeded the ideal body weight, the ratio of actual to ideal body weight was set to 1. Ideal body weight was calculated using the Lorentz formula: for men, WLo = H – 100 – (H – 150)/4; for women, WLo = H – 100 – (H – 150)/2.5, where H represents height (cm) (20).

### 2.3 Variables and outcomes

Fluid balance data from day 1 to day 7 after ICU admission and clinical outcome information were extracted for older patients with malnutrition. Baseline characteristics and relevant clinical variables were also collected, including: (1) demographic characteristics (age, sex, body weight, and height); (2) vital signs (body temperature, heart rate, respiratory rate, and blood pressure); (3) laboratory parameters (complete blood count, blood biochemistry, coagulation profile, blood gas analysis, and lipid profile); (4) comorbidities (heart failure, atrial fibrillation, hypertension, diabetes mellitus, and hyperlipidemia); (5) medication use (diuretics and vasoactive agents); and (6) other clinical information, including the use of continuous renal replacement therapy (CRRT), mechanical ventilation status, and severity scores, such as the Sequential Organ Failure Assessment (SOFA) score and the Charlson Comorbidity Index. All data were extracted from the MIMIC-IV database using structured query language via Navicat Premium 17.0 software. Fluid input and output data consisted of continuous measurements recorded from day 1 to day 7 after ICU admission, while laboratory variables were summarized as mean values within the first 24 h after ICU admission.

Fluid balance was calculated using the following formula: fluid balance = (total fluid intake – total fluid output) / initial body weight (kg). Fluid overload was defined as a cumulative fluid balance exceeding 10% of the initial body weight, calculated as (cumulative fluid balance / initial body weight) × 100% >10% (21). The primary outcome of this study was 30-day in-hospital mortality, defined as survival status within 30 days after ICU admission.

### 2.4 Group-based trajectory model

GBTM was applied to characterize the dynamic trends of fluid balance over time. A cubic polynomial function was used to fit fluid balance data, and models with different numbers of trajectory groups were compared sequentially to determine the optimal number of trajectories. Model performance was comprehensively evaluated based on the following criteria: (1) Bayesian information criterion (BIC), Akaike information criterion (AIC), and entropy, with lower BIC and AIC values and higher entropy indicating better model fit and classification quality; (2) average posterior probability (AvePP) greater than 0.7 to ensure accurate group assignment; (3) a minimum proportion of at least 5% for each trajectory group; (4) odds of correct classification (OCC) with a minimum value greater than 5.0, with higher values indicating better classification performance; and (5) overall consideration of model parsimony and clinical interpretability in addition to statistical adequacy. Although models with a higher number of trajectory groups showed slightly improved information criteria (e.g., lower AIC and BIC values), model selection was not based solely on statistical fit. Instead, a comprehensive evaluation of model parsimony, classification stability, and clinical interpretability was performed. Models with excessive numbers of trajectory groups may result in smaller subgroup sizes and reduced stability, which may limit their clinical applicability. The four-trajectory model was ultimately selected as the optimal model, as it provided a balance between adequate model performance, robust classification quality, and meaningful clinical differentiation, while avoiding over-fragmentation into small and potentially unstable subgroups.

### 2.5 Statistical analysis

All statistical analyses were performed using R software (version 4.4.3; http://www.r-project.org). The normality of continuous variables was assessed using the Shapiro–Wilk test. Normally distributed variables were expressed as mean ± standard deviation and compared using one-way analysis of variance, whereas non-normally distributed variables were presented as median (interquartile range) and compared using the Kruskal–Wallis test. Categorical variables were expressed as counts and percentages and compared using the χ^2^ test or Fisher's exact test, as appropriate. Variables with substantial missing data (e.g., lipid-related variables with missingness exceeding 60%−80%) were excluded to minimize potential bias. For variables with moderate missingness, inclusion was determined based on clinical relevance, and missing values were handled using multiple imputation. Fluid balance variables were not imputed, as trajectory estimation was performed using a group-based trajectory model, which can accommodate incomplete longitudinal data under the missing-at-random assumption. Survival analyses were conducted using Kaplan–Meier curves to describe survival across different fluid balance trajectory groups, and differences were assessed using the log-rank test. Multivariable Cox proportional hazards regression models were further used to evaluate the associations between fluid balance trajectories and 30-day mortality, with results reported as hazard ratios (HRs) and 95% confidence intervals (CIs). Four sequential models were constructed: (1) an unadjusted model; (2) a model adjusted for age and sex; (3) a model further adjusted for vital signs and laboratory variables, including systolic blood pressure, GNRI, serum albumin, creatinine, lactate, and anion gap; and (4) a fully adjusted model additionally including comorbidities and medication use, specifically chronic kidney disease and the use of furosemide, spironolactone, and norepinephrine. Subgroup analyses were performed according to age (65–74 years vs. >74 years), sex (male vs. female), and GNRI category (92–98 indicating mild malnutrition vs. < 92 indicating moderate-to-severe malnutrition) to assess the consistency of the findings. Sensitivity analyses were conducted by excluding patients who received CRRT to evaluate the robustness of the results. All statistical tests were two-sided, and a *P*-value < 0.05 was considered statistically significant.

## 3 Results

### 3.1 Baseline characteristics

**Figure 1:**
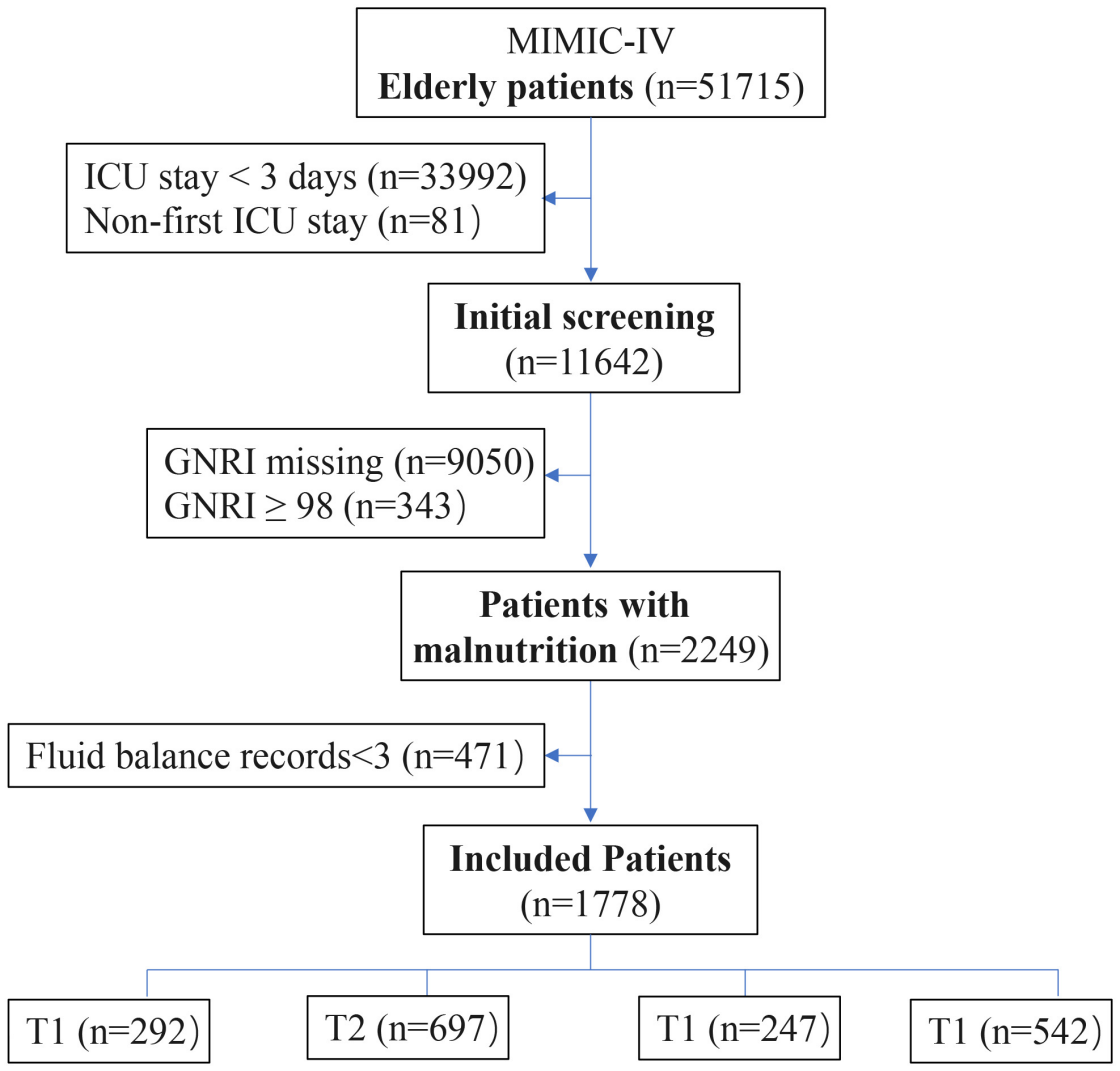
Flowchart of elderly malnourished patient's selection process.

A total of 51,715 older patients were initially screened from the MIMIC-IV database. After applying the predefined exclusion criteria, 2,249 patients with malnutrition were preliminarily identified. Following further data cleaning and completeness assessment, 1,778 older patients with complete clinical data and malnutrition (GNRI < 98) were ultimately included in the analysis (Figure 1). Table 1 summarizes the baseline characteristics of the study population. The median age was 76.0 years, and the median body weight was 78.1 kg. Among the included patients, 991 (55.7%) were male and 787 (44.3%) were female. Regarding comorbidities, 532 patients (29.9%) had chronic kidney disease, and 231 patients (13.0%) received CRRT during their ICU stay. At ICU admission, the median SOFA score was 7.0, and the median Charlson Comorbidity Index was 6.0. The overall 30-day mortality rate was 31.4%.

**Table 1:** Baseline characteristics of elderly malnourished ICU patients (GNRI < 98) stratified by fluid balance trajectory groups.

Variables	Total (*n* = 1,778)	T1 (*n* = 292)	T2 (*n* = 697)	T3 (*n* = 247)	T4 (*n* = 542)	*P*
Demographics						
Age (years)	76.0 [70.0; 82.0]	74.0 [69.0; 81.0]	75.0 [70.0; 82.0]	78.0 [71.0; 84.0]	76.0 [70.0; 82.0]	0.015
Gender						0.005
Female	787 (44.3%)	123 (42.1%)	288 (41.3%)	134 (54.3%)	242 (44.6%)	
Male	991 (55.7%)	169 (57.9%)	409 (58.7%)	113 (45.7%)	300 (55.4%)	
Weight (kg)	78.1 [65.3; 93.5]	78.9 [67.3; 93.8]	82.0 [68.5; 96.1]	70.0 [57.4; 83.7]	77.2 [65.0; 91.9]	< 0.001
Height (cm)	168.0 [160.0; 175.0]	168.0 [160.8; 175.0]	168.0 [160.0; 178.0]	164.0 [157.0; 173.0]	168.0 [160.0; 175.0]	0.001
Vital signs						
T (°C)	36.8 [36.6; 37.2]	36.8 [36.6; 37.2]	36.8 [36.6; 37.1]	36.8 [36.4; 37.2]	36.9 [36.5; 37.2]	0.491
HR (bpm)	85.0 [74.0; 97.0]	87.0 [74.0; 99.0]	81.0 [72.0; 93.0]	92.0 [78.5; 106.0]	85.0 [74.0; 98.0]	< 0.001
RR (bpm)	20.0 [17.0; 23.0]	20.0 [18.0; 23.0]	20.0 [17.0; 22.0]	21.0 [18.0; 24.0]	19.0 [17.0; 22.0]	0.002
SBP (mmHg)	110.0 [102.0; 122.0]	110.0 [102.0; 120.0]	115.0 [105.0; 129.0]	106.0 [99.0; 111.0]	109.0 [101.0; 120.0]	< 0.001
DBP (mmHg)	60.0 [54.0; 68.0]	60.5 [53.0; 69.0]	62.0 [55.0; 69.0]	58.0 [51.0; 65.0]	59.0 [53.2; 67.0]	< 0.001
MBP (mmHg)	73.0 [67.0; 81.0]	73.0 [66.0; 81.0]	75.0 [69.0; 83.0]	69.0 [64.0; 76.0]	72.0 [66.0; 79.0]	< 0.001
SpO2 (%)	97.0 [96.0; 99.0]	97.0 [95.0; 98.2]	97.0 [95.0; 98.0]	98.0 [96.0; 99.0]	98.0 [96.0; 99.0]	< 0.001
Laboratory indicators						
WBC (m/uL)	11.8 [8.3; 16.8]	11.4 [8.3; 15.5]	11.0 [7.9; 15.3]	13.6 [7.9; 20.0]	12.6 [8.7; 17.7]	< 0.001
PLT (K/uL)	185.0 [130.0; 259.0]	184.5 [125.8; 255.5]	192.0 [132.0; 264.0]	186.0 [120.5; 262.0]	176.0 [130.0; 254.0]	0.402
Hb (g/dL)	10.3 [8.9; 11.9]	10.2 [8.8; 11.8]	10.4 [8.9; 11.9]	10.5 [9.3; 12.1]	10.2 [8.6; 11.7]	0.047
RDW (%)	15.0 [13.9; 16.5]	15.2 [14.0; 16.8]	15.0 [14.0; 16.5]	14.9 [13.8; 16.6]	14.8 [13.8; 16.3]	0.169
HCT (%)	31.9 [27.5; 36.3]	31.4 [27.1; 35.6]	32.1 [27.6; 36.4]	32.8 [28.7; 37.4]	31.3 [27.2; 36.1]	0.022
Na (mEq/L)	139.0 [135.0; 142.0]	138.0 [134.0; 141.0]	139.0 [135.0; 142.0]	140.0 [136.0; 143.0]	139.0 [136.0; 142.0]	0.004
K (mEq/L)	4.2 [3.8; 4.7]	4.2 [3.8; 4.9]	4.2 [3.8; 4.7]	4.2 [3.7; 4.8]	4.2 [3.8; 4.7]	0.408
Ca (mg/dL)	8.1 [7.6; 8.7]	8.1 [7.7; 8.7]	8.4 [7.9; 8.8]	7.6 [7.0; 8.2]	8.1 [7.5; 8.5]	< 0.001
Cl (mEq/L)	104.0 [100.0; 109.0]	103.0 [98.0; 108.0]	103.0 [99.0; 107.0]	108.0 [103.0; 112.0]	106.0 [101.2; 110.0]	< 0.001
Glu (mg/dL)	137.0 [109.0; 184.0]	141.0 [108.0; 185.2]	133.0 [108.0; 180.0]	138.0 [108.5; 183.0]	140.0 [112.0; 190.5]	0.384
AG (mEq/L)	15.0 [12.0; 18.0]	16.0 [13.0; 19.0]	15.0 [12.0; 17.0]	16.0 [13.0; 19.0]	15.0 [12.0; 18.0]	< 0.001
ALT (IU/L)	28.0 [16.0; 66.8]	31.0 [16.0; 82.0]	27.0 [16.0; 60.0]	33.0 [17.5; 89.0]	27.0 [16.0; 56.8]	0.062
AST (IU/L)	45.0 [26.0; 108.0]	52.0 [27.0; 140.2]	40.0 [25.0; 86.0]	56.0 [30.0; 149.5]	45.0 [25.2; 101.0]	< 0.001
BUN (mg/dL)	28.0 [18.0; 45.0]	31.5 [19.8; 53.2]	28.0 [18.0; 46.0]	30.0 [18.0; 45.5]	25.0 [17.0; 40.0]	< 0.001
Cr (mg/dL)	1.2 [0.9; 2.0]	1.6 [0.9; 2.8]	1.2 [0.9; 1.8]	1.3 [0.9; 1.9]	1.1 [0.8; 1.7]	< 0.001
pH	7.4 [7.3; 7.4]	7.3 [7.3; 7.4]	7.4 [7.3; 7.4]	7.3 [7.2; 7.4]	7.3 [7.3; 7.4]	< 0.001
PO2 (mmHg)	91.0 [57.0; 176.8]	92.0 [60.8; 175.2]	87.0 [54.0; 158.0]	89.0 [55.0; 174.5]	98.0 [60.0; 218.2]	0.033
PCO2 (mmHg)	41.0 [34.0; 48.0]	40.0 [34.0; 48.0]	41.0 [35.0; 48.0]	40.0 [33.5; 47.0]	40.0 [34.0; 47.0]	0.190
Lac (mmol/L)	1.8 [1.2; 2.9]	1.8 [1.2; 2.9]	1.5 [1.1; 2.2]	2.5 [1.5; 4.3]	1.9 [1.3; 3.3]	< 0.001
PT (sec)	14.8 [13.0; 18.0]	15.1 [13.1; 19.0]	14.0 [12.7; 17.1]	15.2 [13.5; 17.8]	15.0 [13.3; 18.6]	< 0.001
APTT (sec)	32.5 [28.0; 42.6]	34.4 [28.5; 44.8]	32.0 [27.7; 42.1]	33.4 [29.2; 42.6]	32.0 [27.8; 41.6]	0.069
INR (ratio)	1.3 [1.2; 1.7]	1.4 [1.2; 1.7]	1.3 [1.1; 1.6]	1.4 [1.2; 1.6]	1.4 [1.2; 1.7]	< 0.001
GNRI	84.9 [78.9; 90.8]	84.9 [78.2; 90.6]	87.9 [81.9; 92.3]	80.3 [74.5; 84.9]	84.9 [78.9; 89.5]	< 0.001
Comorbidities						
HF, *N* (%)	758 (42.6%)	125 (42.8%)	380 (54.5%)	66 (26.7%)	187 (34.5%)	< 0.001
AF, *N* (%)	842 (47.4%)	154 (52.7%)	335 (48.1%)	107 (43.3%)	246 (45.4%)	0.112
Hypertension, *N* (%)	1,340 (75.4%)	212 (72.6%)	539 (77.3%)	173 (70.0%)	416 (76.8%)	0.072
DM, *N* (%)	323 (18.2%)	49 (16.8%)	133 (19.1%)	41 (16.6%)	100 (18.5%)	0.751
Hyperlipidemia, *N* (%)	783 (44.0%)	123 (42.1%)	346 (49.6%)	92 (37.2%)	222 (41.0%)	0.001
COPD, *N* (%)	182 (10.2%)	31 (10.6%)	87 (12.5%)	11 (4.5%)	53 (9.8%)	0.005
Pneumonia, *N* (%)	292 (16.4%)	43 (14.7%)	108 (15.5%)	55 (22.3%)	86 (15.9%)	0.062
CKD, *N* (%)	532 (29.9%)	94 (32.2%)	245 (35.2%)	60 (24.3%)	133 (24.5%)	< 0.001
Cirrhosis, *N* (%)	118 (6.6%)	30 (10.3%)	45 (6.5%)	6 (2.4%)	37 (6.8%)	0.004
Sepsis, *N* (%)	1,515 (85.2%)	274 (93.8%)	536 (76.9%)	236 (95.5%)	469 (86.5%)	< 0.001
Medications						
Furosemide	1,432 (80.5%)	214 (73.3%)	563 (80.8%)	201 (81.4%)	454 (83.8%)	0.004
Spironolactone	109 (6.1%)	18 (6.2%)	52 (7.5%)	6 (2.4%)	33 (6.1%)	0.045
Dobutamine	100 (5.6%)	27 (9.2%)	45 (6.5%)	13 (5.3%)	15 (2.8%)	0.001
Dopamine	137 (7.7%)	33 (11.3%)	45 (6.5%)	24 (9.7%)	35 (6.5%)	0.024
Epinephrine	202 (11.4%)	48 (16.4%)	47 (6.7%)	25 (10.1%)	82 (15.1%)	< 0.001
Norepinephrine	1,006 (56.6%)	218 (74.7%)	288 (41.3%)	189 (76.5%)	311 (57.4%)	< 0.001
Phenylephrine	745 (41.9%)	159 (54.5%)	198 (28.4%)	158 (64.0%)	230 (42.4%)	< 0.001
Other indicators						
Ventilation	1,681 (94.5%)	272 (93.2%)	651 (93.4%)	242 (98.0%)	516 (95.2%)	0.030
CRRT	231 (13.0%)	128 (43.8%)	36 (5.2%)	49 (19.8%)	18 (3.3%)	< 0.001
SOFA	7.0 [4.0; 10.0]	8.0 [5.0; 11.0]	6.0 [3.0; 9.0]	9.0 [6.0; 11.0]	8.0 [5.0; 10.0]	< 0.001
Charlson	6.0 [5.0; 8.0]	7.0 [5.0; 8.0]	7.0 [5.0; 9.0]	6.0 [4.0; 8.0]	6.0 [5.0; 8.0]	< 0.001
30-day mortality	558 (31.4%)	135 (46.2%)	196 (28.1%)	83 (33.6%)	144 (26.6%)	< 0.001

ICU, intensive care unit; GNRI, Geriatric Nutritional Risk Index; T, temperature; HR, heart rate; RR, respiratory rate; SBP, systolic blood pressure; DBP, diastolic blood pressure; MBP, mean blood pressure; SpO_2_, peripheral oxygen saturation; WBC, white blood cell count; PLT, platelet count; Hb, hemoglobin; RDW, red cell distribution width; HCT, hematocrit; Na, sodium; K, potassium; Ca, calcium; Cl, chloride; Glu, glucose; AG, anion gap; ALT, alanine aminotransferase; AST, aspartate aminotransferase; BUN, blood urea nitrogen; Cr, creatinine; pH, potential of hydrogen; PO_2_, partial pressure of oxygen; PCO_2_, partial pressure of carbon dioxide; Lac, lactate; PT, prothrombin time; APTT, activated partial thromboplastin time; INR, international normalized ratio; HF, heart failure; AF, atrial fibrillation; DM, diabetes mellitus; COPD, chronic obstructive pulmonary disease; CKD, chronic kidney disease; CRRT, continuous renal replacement therapy; SOFA, Sequential Organ Failure Assessment score; Charlson, Charlson Comorbidity Index.

### 3.2 Description of fluid balance trajectories

The model fit indices for different numbers of trajectory groups are presented in Table 2. As the number of trajectory groups increased, the BIC and AIC values progressively decreased, indicating improved model fit. The four-trajectory model demonstrated marked reductions in information criteria while maintaining good classification performance, with average posterior probabilities exceeding 0.80 for all trajectory groups and a minimum odds of correct classification of 11.51, indicating high classification accuracy and stability. In addition, the entropy value of the four-trajectory model was 0.76, suggesting good discrimination between trajectory groups. Although the five-trajectory model showed slightly improved information criteria (AIC and BIC), it was associated with increased model complexity and smaller subgroup proportions, which may reduce stability and limit clinical interpretability. Therefore, the four-trajectory model was selected based on a balance between model fit, classification performance, and clinical interpretability. Considering model fit, classification stability, and clinical interpretability, the four-trajectory model was ultimately selected for subsequent analyses.

**Table 2:** Performance of the group-based trajectory model for fluid balance trajectories.

Trajectories	BIC	AIC	AvePP	Entropy	Minimum OCC	Class proportion
1 group	117,946	117,909	1.00	NaN	NaN	100.0%
2 groups	114,894	114,812	0.93/0.94	0.79	10.84	39.2%/60.8%
3 groups	114,375	114,248	0.84/0.94/0.91	0.82	11.04	13.2%/60.4%/26.4%
4 groups	113,763	113,599	0.85/0.89/0.92/0.83	0.76	11.51	16.0%/39.5%/14.0%/30.5%
5 groups	113,205	112,990	0.87/0.85/0.93/0.84/0.83	0.77	13.46	13.0%/30.4%/16.3%/13.6%/26.8%

BIC, Bayesian information criterion; AIC, Akaike information criterion; AvePP, average posterior probability; Occ, odds of correct classification; NAN, not available.

**Figure 2:**
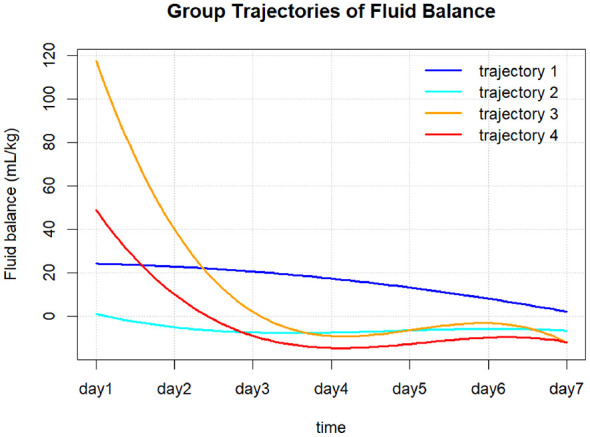
Fluid balance trajectories in elderly malnourished patients.

As shown in Figure 2, trajectory analysis based on fluid balance identified four distinct patterns of fluid balance change (T1–T4). T1 (persistent positive balance) represented a persistent positive balance pattern, in which patients maintained a moderate positive fluid balance of approximately 20 mL/kg after ICU admission, followed by a gradual and slow decline over time, while remaining positive throughout the observation period. T2 (mild negative balance) represented a mild negative balance pattern, in which patients were close to zero balance early after ICU admission and gradually transitioned to a mild negative balance of approximately −5 mL/kg, remaining relatively stable during follow-up. T3 (high-level rapid decline) was characterized as a high-level rapid decline pattern, with patients exhibiting marked positive fluid balance on day 1 after ICU admission (approximately 120 mL/kg), followed by a rapid decrease to near-zero or mildly negative levels within the first 3 days, after which changes became more gradual. T4 (moderate-level rapid decline) represented a moderate-level rapid decline pattern, in which patients initially presented with a moderate positive fluid balance of approximately 50 mL/kg, followed by a rapid decline and maintenance of a negative balance after days 3–4.

The 30-day mortality rate was highest in T1 (46.2%), followed by T3 (33.6%), T2 (28.1%), and T4 (26.6%). As shown in Table 1, significant differences in multiple baseline characteristics were observed among T1–T4. Patients in the T3 group were older, had lower body weight, higher heart rate and respiratory rate, lower blood pressure at ICU admission, and higher inflammatory markers, lactate levels, and SOFA scores. The T1 group exhibited poorer renal function indicators and the highest proportions of continuous renal replacement therapy and vasoactive drug use. In contrast, the T2 group had the lowest SOFA scores and a lower proportion of invasive supportive therapies. Baseline characteristics in the T4 group were generally intermediate between those of the T1 and T3 groups.

### 3.3 Associations of fluid balance trajectories and fluid overload with survival

**Figure 3:**
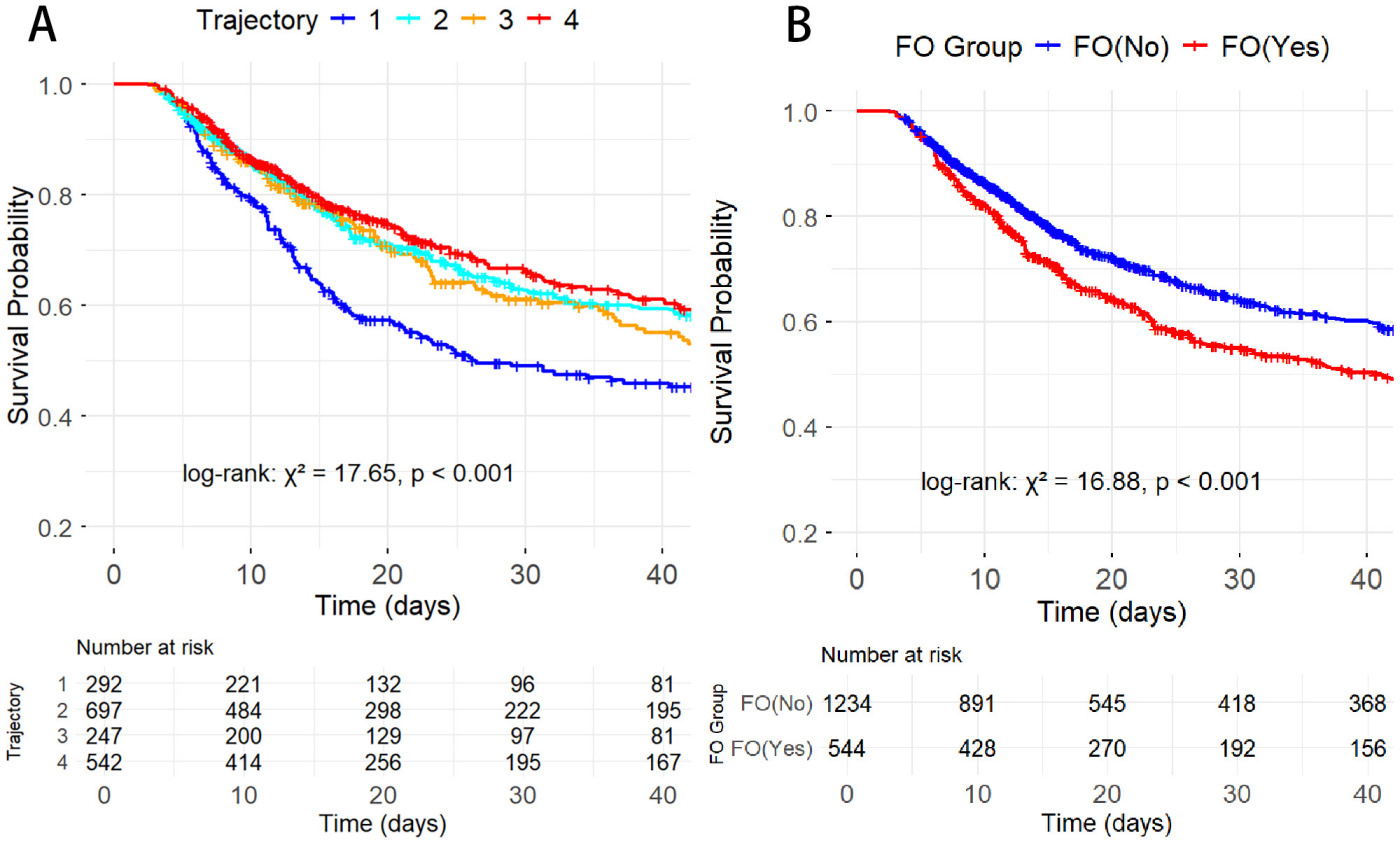
Kaplan–Meier curves of 30-day mortality by fluid balance trajectories **(A)** and fluid overload status **(B)** in elderly malnourished patients.

Kaplan–Meier survival analyses demonstrated significant differences in survival among the fluid balance trajectory groups (*P* < 0.001), as shown in Figure 3. Patients in T4, T2, and T3 groups had better survival compared with those in T1. In addition, patients with fluid overload had a significantly higher risk of mortality than those without fluid overload (*P* < 0.001).

Multiple Cox proportional hazards models were constructed with stepwise adjustment for potential confounders, and Table 3 presents the associations between fluid balance trajectories and 30-day mortality. In the unadjusted model (Model 1), the risks of mortality were significantly lower in the T2 and T4 groups compared with the T1 group, whereas no statistically significant difference was observed for the T3 group. After adjustment for age and sex (Model 2), these associations remained largely unchanged. With further adjustment for hemodynamic parameters, nutritional status, and metabolic-related variables in Model 3, the risks of mortality in the T2, T3, and T4 groups were all significantly lower than that in the T1 group, with the association for the T4 group being the most consistent. In the fully adjusted model (Model 4), the T4 group remained significantly associated with a lower risk of 30-day mortality, whereas the risk reductions observed in the T2 and T3 groups were attenuated and no longer statistically significant.

**Table 3:** Cox regression multimodel analysis of FB trajectories in elderly malnourished patients (GNRI < 98).

Variables	Model 1		Model 2		Model 3		Model 4	
	**HR (95%CI)**	***P***	**HR (95%CI)**	***P***	**HR (95%CI)**	***P***	**HR (95%CI)**	***P***
Trajectory
T1	1.00 (Reference)		1.00 (Reference)		1.00 (Reference)		1.00 (Reference)	
T2	0.74 (0.62–0.87)	< 0.001	0.71 (0.60–0.84)	< 0.001	0.77 (0.64–0.92)	0.003	0.84 (0.70–1.01)	0.063
T3	0.87 (0.71–1.07)	0.196	0.84 (0.69–1.04)	0.105	0.79 (0.64–0.98)	0.032	0.82 (0.66–1.02)	0.073
T4	0.72 (0.60–0.86)	< 0.001	0.70 (0.59–0.84)	< 0.001	0.72 (0.60–0.86)	< 0.001	0.76 (0.63–0.91)	0.003

HR, Hazard Ratio; CI, Confidence Interval.

Model 1: Crude.

Model 2: Adjust: Age, Gender.

Model 3: Adjust: Age, Gender, SBP, GNRI, ALB, Cr, Lac, AG.

Model 4: Adjust: Age, Gender, SBP, GNRI, ALB, Cr, Lac, AG, CKD, Furosemide, Spironolactone, Norepinephrine.

### 3.4 Subgroup analyses

**Figure 4:**
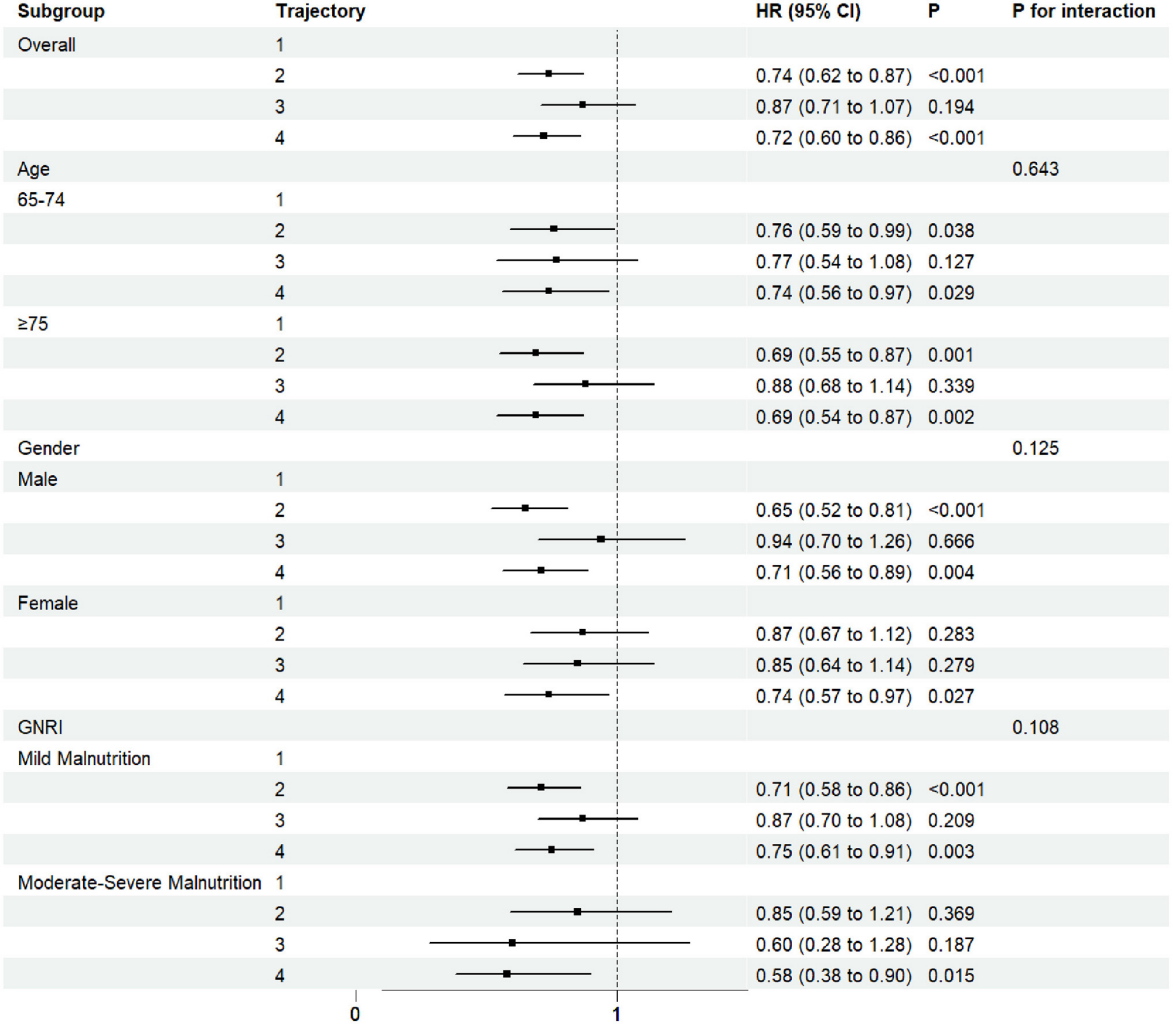
Forest subgroup analysis FB Trajectories and 30-day ICU mortality in elderly malnourished patients.

The results of subgroup analyses are shown in Figure 4. Across stratifications by age, sex, and degree of malnutrition, the direction of the association between fluid balance trajectories and 30-day mortality was generally consistent. Compared with the T1 group, the T4 group was significantly associated with a lower risk of mortality in most subgroups, with a more pronounced risk reduction observed among patients with moderate-to-severe malnutrition. The T2 group was significantly associated with reduced mortality risk among male patients and across different age strata, but did not reach statistical significance among female patients or those with moderate-to-severe malnutrition. No significant associations were observed for the T3 trajectory across subgroups. No statistically significant interactions were detected among subgroups.

### 3.5 Sensitivity analyses

**Figure 5:**
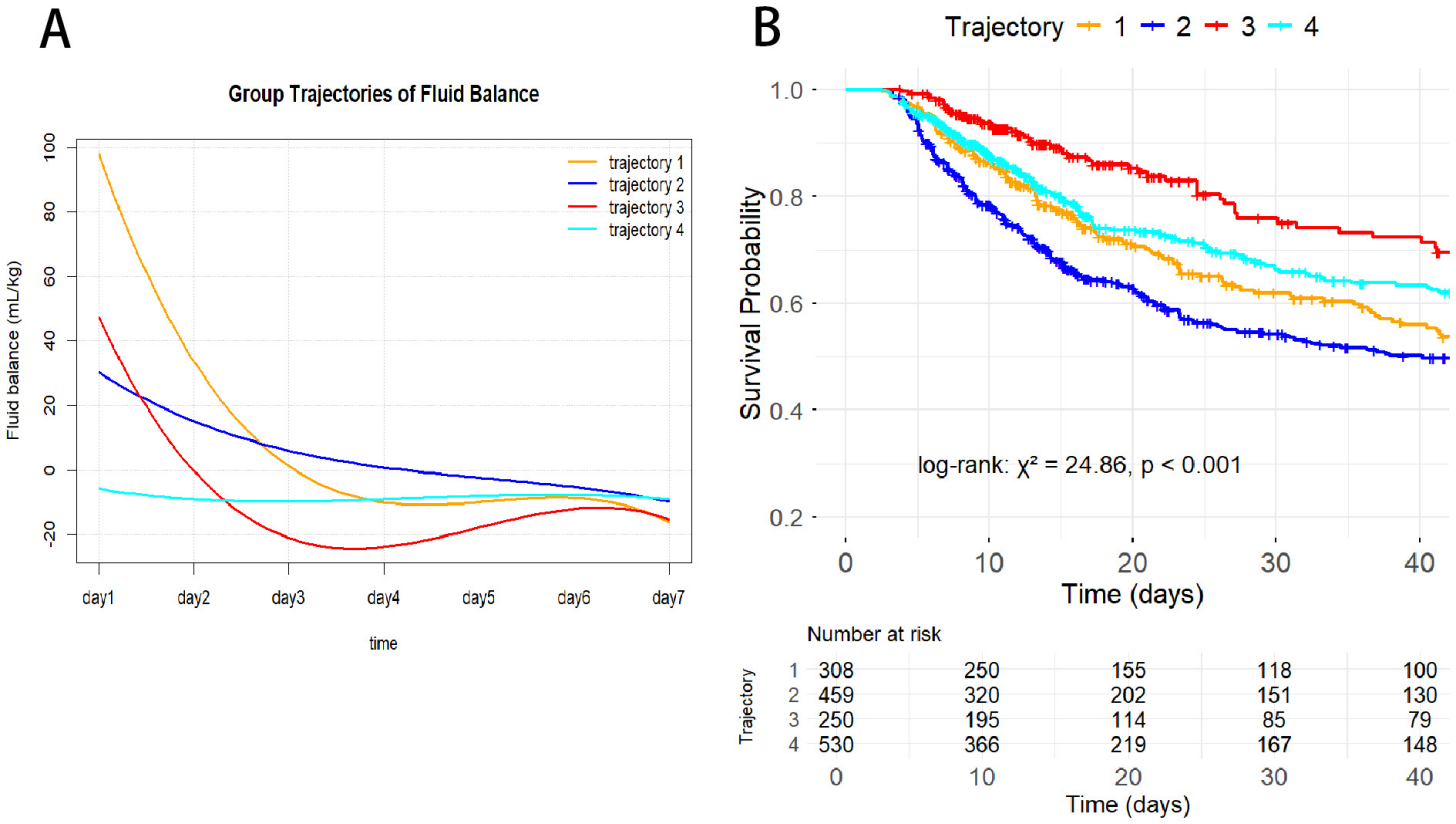
Sensitivity analysis excluding CRRT in elderly malnourished patients **(A)** fluid balance trajectories; **(B)** Kaplan–Meier survival curves.

Sensitivity analyses further confirmed the robustness of the association between fluid balance trajectories and prognosis in older patients with malnutrition. After excluding patients who received CRRT, 1,547 patients were included in the sensitivity analysis, and the fluid balance trajectory patterns remained consistent with those identified in the primary analysis (Figure 5A).

Kaplan–Meier analyses demonstrated significant differences in 30-day survival among the trajectory groups (log-rank test, *P* < 0.001; Figure 5B). Notably, the moderate-level rapid decline group remained significantly associated with a lower risk of 30-day all-cause mortality compared with the persistent positive balance group, consistent with the primary findings and further supporting the stability of the results.

## 4 Discussion

Based on the MIMIC-IV database, this study applied a group-based trajectory model to characterize dynamic patterns of fluid balance in older ICU patients with malnutrition and to evaluate the associations between different fluid balance trajectories and 30-day mortality. Four distinct fluid balance trajectories with clear clinical features were identified. The results demonstrated significant differences in 30-day mortality risk across these trajectories. After comprehensive multivariable adjustment, the moderate-level rapid decline trajectory remained significantly associated with a lower risk of 30-day mortality compared with the persistent positive balance trajectory. This association was consistent across subgroup analyses and sensitivity analyses excluding patients receiving CRRT. In addition, fluid overload was independently associated with an increased risk of mortality.

Previous studies have consistently shown that fluid overload or persistent positive fluid balance is closely associated with increased mortality in ICU patients (22). Our findings are concordant with these observations, with adverse outcomes predominantly observed among patients exhibiting high-level or persistent positive fluid balance trajectories. Notably, patients in the persistent positive balance group (T1) had higher rates of CRRT use and vasopressor support, suggesting a greater burden of hemodynamic instability and renal dysfunction. Therefore, this trajectory pattern may, at least in part, reflect an underlying disease severity phenotype characterized by refractory shock and impaired fluid handling, rather than solely a treatment-related effect. Although adjustments were made for multiple confounders, including SOFA score, CRRT, and vasopressor use, residual confounding by indication cannot be excluded. Thus, the association between persistent positive fluid balance and mortality should be interpreted cautiously, as it may represent both impaired physiological status and treatment resistance. Systematic reviews have reported that, in critically ill adults, fluid overload—defined either by cumulative fluid balance or weight gain—is significantly associated with increased mortality (8). Multicenter cohort studies have further demonstrated that sustained high positive or cumulative fluid balance is significantly associated with in-hospital and 28- or 30-day mortality in ICU subpopulations such as acute kidney injury, sepsis, and aortic dissection (10, 23, 24). Unlike these studies, which primarily relied on static analyses based on single time points or fixed time windows, the present study employed dynamic trajectory analysis to more accurately capture the processes of fluid administration and deresuscitation. A limited number of studies using trajectory-based approaches have similarly reported that persistent positive balance trajectories are associated with higher in-hospital or 90-day mortality among patients with septic shock or those receiving renal replacement therapy, suggesting that dynamic trajectories effectively discriminate prognostic risk (21, 25). However, most prior investigations focused on general ICU populations or specific disease cohorts, with limited attention to the vulnerable subgroup of older patients with malnutrition. By focusing on this population, the present study extends existing evidence and provides novel insights into the relationship between fluid balance dynamics and prognosis.

Our findings indicate that the moderate-level rapid decline trajectory was significantly associated with a lower risk of 30-day mortality, suggesting that early fluid resuscitation may be associated with improved hemodynamic stability, whereas excessive fluid loading may be associated with adverse outcomes. Similar observations have been reported in patients with septic shock (21). These results support a fluid management strategy in which a certain degree of positive balance is acceptable during early resuscitation, followed by a timely transition to the deresuscitation phase to avoid fluid overload and excessive cardiac and renal burden. Fluid management in older patients with malnutrition is particularly complex due to age-related physiological changes and disease-related factors. With aging, total body water decreases, muscle mass declines, and renal concentrating capacity is impaired, substantially reducing the ability to regulate fluid balance (26). In addition, malnutrition-related losses of muscle mass and serum albumin further diminish intracellular water reserves and oncotic pressure, predisposing patients to pronounced fluctuations in intravascular and interstitial fluid volumes during fluid administration or loss (27). In the setting of fluid overload, fluid retention may increase cardiac preload and renal burden and may be associated with worsening hypoperfusion and organ dysfunction (28). These considerations highlight the importance of integrating fluid and nutritional management and tailoring strategies according to individual patient phenotypes and trajectory patterns.

The strengths of this study include the use of a dynamic fluid balance trajectory approach, which more accurately reflects temporal changes in fluid status and overcomes the limitations of traditional static or cumulative measures. By capturing longitudinal fluid balance patterns, this approach enhances prognostic assessment and provides an efficient, low-cost tool to inform clinical fluid management. In the ICU setting, fluid balance trajectory analysis may assist clinicians in optimizing fluid strategies, particularly in older patients with malnutrition, by improving risk stratification and facilitating individualized treatment planning. Future prospective studies are warranted to validate the causal relationship between fluid balance trajectories and mortality and to account for variations in fluid management practices across different ICU settings, thereby promoting broader clinical application of this approach and improving the management of high-risk patients.

Several limitations should be acknowledged. First, this study was based on a single-center database, which may introduce selection bias, and its retrospective design precludes causal inference between fluid balance trajectories and mortality; prospective studies are required to confirm these findings. Second, patients with ICU stays of less than 3 days and those with insufficient fluid balance measurements were excluded, which may have introduced selection bias. In particular, patients who died early after ICU admission may have been systematically excluded, potentially leading to an underrepresentation of the most severely ill individuals. This may have resulted in an underestimation of overall mortality risk and may have attenuated the observed associations between fluid balance trajectories and mortality. In addition, patients with incomplete fluid balance data may differ in clinical characteristics or management intensity, further contributing to potential bias. Third, the use of repeated fluid balance measurements over the first 7 ICU days to define trajectory groups may introduce a potential risk of immortal time bias, as patients must survive long enough to be classified. Although the exposure window preceded outcome assessment and trajectory modeling incorporated all available observations within this period, this bias cannot be entirely excluded. Fourth, despite adjustment for multiple confounders, residual confounding cannot be excluded, as factors such as diuretic dosage and echocardiographic parameters were not available and may have affected the accuracy of the results. In addition, detailed information on nutritional fluid intake, such as enteral or parenteral nutrition (EN/PN), was not separately available in the database. Therefore, the contribution of nutritional support to total fluid balance could not be specifically quantified. Given that the study population consisted of malnourished patients, nutritional interventions may have influenced fluid balance trajectories, potentially introducing additional confounding. Fifth, multiple imputation and model-based approaches were used to handle missing data, some degree of uncertainty remains. In particular, the absence of complete-case analysis may limit the assessment of robustness, and the findings should be interpreted with caution. Finally, fluid balance trajectories may partly reflect underlying disease severity rather than solely treatment strategies. Although adjustments were made for multiple confounders, including SOFA score and other clinical variables, residual confounding by indication cannot be excluded. Patients with more severe illness may be more likely to receive aggressive fluid resuscitation or exhibit impaired fluid regulation, which may influence both fluid balance patterns and outcomes. Therefore, the observed associations should be interpreted with caution and should not be considered as evidence of causal relationships. Future studies may explore more granular trajectory classifications to further characterize heterogeneity.

## 5 Conclusion

Using a group-based trajectory modeling approach, this study demonstrated a significant association between fluid balance trajectories and 30-day mortality in older patients with malnutrition. A moderate-level rapid decline trajectory was independently associated with a lower risk of mortality, suggesting that early fluid resuscitation may be associated with hemodynamic stabilization, whereas excessive fluid loading may be associated with adverse outcomes. These findings highlight the clinical value of fluid balance trajectory analysis as a prognostic assessment tool. Further prospective and randomized studies are needed to establish causal relationships and to validate these findings.

## Data Availability

The original contributions presented in the study are included in the article/supplementary material, further inquiries can be directed to the corresponding author.
